# Early Development of Ubiquitous Acanthocytosis and Extravascular Hemolysis in Lung Cancer Patients Receiving Alectinib

**DOI:** 10.3390/cancers14112720

**Published:** 2022-05-31

**Authors:** Julia Kunz, Christiane Wiedemann, Heidrun Grosch, Katharina Kriegsmann, Stefanie Gryzik, Julia Felden, Michael Hundemer, Huriye Seker-Cin, Miriam Stenzinger, Albrecht Leo, Albrecht Stenzinger, Michael Thomas, Petros Christopoulos

**Affiliations:** 1 Department of Thoracic Oncology, Heidelberg University Hospital, 69126 Heidelberg, Germany; julia.kunz@med.uni-heidelberg.de (J.K.); christiane.wiedemann@med.uni-heidelberg.de (C.W.); heidrun.grosch@med.uni-heidelberg.de (H.G.); michael.thomas@med.uni-heidelberg.de (M.T.); 2 Department of Internal Medicine V (Hematology/Oncology/Rheumatology), Heidelberg University Hospital, 69120 Heidelberg, Germany; katharina.kriegsmann@med.uni-heidelberg.de (K.K.); stefanie.grycik@med.uni-heidelberg.de (S.G.); michael.hundemer@med.uni-heidelberg.de (M.H.); 3 Hematology Laboratory MVZ, 64646 Heppenheim, Germany; julia.felden@med.uni-heidelberg.de; 4 Institute of Pathology, Heidelberg University Hospital, 69120 Heidelberg, Germany; huriye.seker-cin@med.uni-heidelberg.de (H.S.-C.); albrecht.stenzinger@med.uni-heidelberg.de (A.S.); 5 Institute of Immunology, Heidelberg University Hospital, 69120 Heidelberg, Germany; miriam.stenzinger@med.uni-heidelberg.de (M.S.); albrecht.leo@med.uni-heidelberg.de (A.L.); 6 Translational Lung Research Center Heidelberg (TLRC-H), Member of the German Center for Lung Research (DZL), 69120 Heidelberg, Germany

**Keywords:** ALK+ NSCLC, alectinib, anemia, hemolysis, acanthocytosis

## Abstract

**Simple Summary:**

ALK translocation is present in 4–5% of patients with non-small-cell lung cancer. Patients treated with alectinib invariably develop subclinical hemolysis, which is presumably caused by acanthocytic deformation and splenic trapping of blood erythrocytes. Morphologic red blood cell changes, decreased EMA testing, and reactive reticulocytosis develop in literally all patients early after the initiation of alectinib, but not other ALK inhibitors. These alterations are not associated with the efficacy of alectinib or the molecular features of ALK+ disease and resolve within a few months after a switch to other ALK–TKI but complicate any hematologic workup. Furthermore, the risk of long-term complications, such as cholelithiasis due to increased serum bilirubin in the majority of cases, remains unclear at present.

**Abstract:**

Alectinib is a standard initial treatment for patients with advanced anaplastic lymphoma kinase (ALK) rearranged non-small-cell lung cancer (NSCLC). The current study analyzed a prospective cohort of 24 consecutive alectinib-treated patients and controls in order to comprehensively characterize longitudinal erythrocyte changes under treatment with ALK inhibitors. Upon starting alectinib, all examined patients developed reticulocytosis and abnormal erythrocyte morphology with anisocytosis and a predominance of acanthocytes (64% of red blood cells on average, range 36–100%) in the peripheral blood smear within approximately 2 weeks. Changes were accompanied by a gradual reduction in Eosin-5-maleimide (EMA) binding, which became pathologic (<80% of cells) within 1–2 months in all cases, mimicking an abortive form of hereditary spherocytosis. The latter could be ruled out in 3/3 of analyzed cases by normal sequencing results for the *ANK1*, *EPB42*, *SLC4A1*, *SPTA1*, or *SBTB* genes. The direct Coombs test was also negative in 11/11 tested cases. Besides, anemia, increased LDH, and increased bilirubin were noted in a fraction of patients only, ranging between 42 and 68%. Furthermore, haptoglobin decreases were infrequent, occurring in approximately 1/3 of cases only, and mild, with an average value of 0.93 g/L within the normal range of 0.3–2 g/dL, suggesting that hemolysis occurred predominantly in the extravascular compartment, likely due to splenic trapping of the deformed erythrocytes. These changes showed no association with progression-free survival under alectinib or molecular features, i.e., *ALK* fusion variant or *TP53* status of the disease, and resolved upon a switch to an alternative ALK inhibitor. Thus, alectinib induces mild, reversible erythrocyte changes in practically all treated patients, whose most sensitive signs are aberrant red cell morphology in the peripheral smear, a pathologic EMA test, and reactive reticulocytosis. Frank hemolytic anemia is rare, but mild subclinical hemolysis is very frequent and poses differential-diagnostic problems. Alectinib can be continued under the regular control of hemolysis parameters, but the risk of long-term complications, such as cholelithiasis due to increased serum bilirubin in most patients, remains unclear at present.

## 1. Introduction

Alectinib is a highly selective, second-generation tyrosine kinase inhibitor (TKI) of the anaplastic lymphoma kinase (ALK), which is constitutively activated via gene translocation and serves as the driver oncogene in approximately 4–5% of non-small-cell lung cancers (NSCLC) [1]. The higher systemic and brain efficacy of alectinib and other next-generation ALK inhibitors compared to crizotinib translates to significant survival benefits, making these newer substances the current standard initial treatment for newly-diagnosed advanced ALK+ NSCLC patients [2,3,4,5,6,7]. The emerging therapeutic landscape of ALK+ disease has been comprehensively reviewed recently [8]. While the wide adoption of the “best-first” approach was a major step forward, pushing the median survival of stage IV over the 5-year limit [9], caution is warranted for potential long-term risks. For alectinib, tolerability is generally very good, with mostly grade 1/2 adverse effects such as fatigue, constipation, peripheral edema, and myalgia. More frequent grade 3 or 4 reactions include increases in creatine phosphokinase, alanine aminotransferase, and aspartate aminotransferase levels, while grade 3–4 anemia is rare (≤5%) [10]. The current study was triggered by the incidental observation of hemolysis signs in several alectinib-treated patients, and its aim was to comprehensively characterize red blood cell changes under this drug.

## 2. Materials and Methods

Between August 2020 and August 2021, we recruited 19 patients treated consecutively with alectinib for metastatic ALK+ NSCLC in various lines at the Thoraxklinik Heidelberg. These patients underwent laboratory, blood morphologic, and molecular analysis in order to characterize the properties of alectinib-induced hemolysis. In addition, five patients with advanced ALK+ NSCLC receiving other ALK TKIs (crizotinib, brigatinib, lorlatinib) were analyzed as controls. Histologic diagnosis of NSCLC was based on the 2015 classification of thoracic malignancies by the World Health Organization [11], while molecular tumor workup was based on semiconductor-based combined DNA and RNA next-generation sequencing (NGS) using a 40-gene panel, including typing of the *ALK* fusion variant and *TP53* status, as published [12]. For functional annotation of detected *TP53* mutations, the COSMIC, ClinVar, OncoKB, and Jackson Laboratory databases, as well as the current literature, were consulted [13]. For the characterization of red blood cell changes, routine laboratory tests included complete blood counts with differential, indirect bilirubin, reticulocyte counts, haptoglobin, and lactate dehydrogenase (LDH). Peripheral blood smears were analyzed by expert hematologists in 12 cases, while the direct antiglobulin (Coombs) test was performed in 11 patients. In addition, eosin-5-maleimide (EMA) assays, which measure the fluorescence intensity of erythrocytes washed and incubated with the membrane-intercalating, band-3 binding agent EMA [14], were performed in 12 cases, and genetic testing for hereditary spherocytosis through DNA NGS of the *ANK1*, *EPB42*, *SLC4A1*, *SPTA1*, or *SBTB* genes was undertaken in 3 cases [15]. Clinical data were collected from the patients’ records. Overall survival (OS) was calculated from the diagnosis of stage IV disease. The duration of follow-up was calculated using the reverse Kaplan–Meier method. For progression-free survival (PFS), the progression date was collected from the records and verified with a review of radiologic images, i.e., chest CT/brain MRI every 6–12 weeks, by the investigators without formal RECIST re-evaluation, as several studies have demonstrated very good agreement between real-world and RECIST-based assessments [16,17]. The association of survival with numerical variables was analyzed with a Cox regression while examining the potential difference in red blood cell and hemolysis parameters according to the molecular features of the disease, i.e., *EML4-ALK* variant status (V3 or non-V3) and *TP53* status (wild-type or mutated); unpaired *t*-tests were used.

## 3. Results

**Figure 1:**
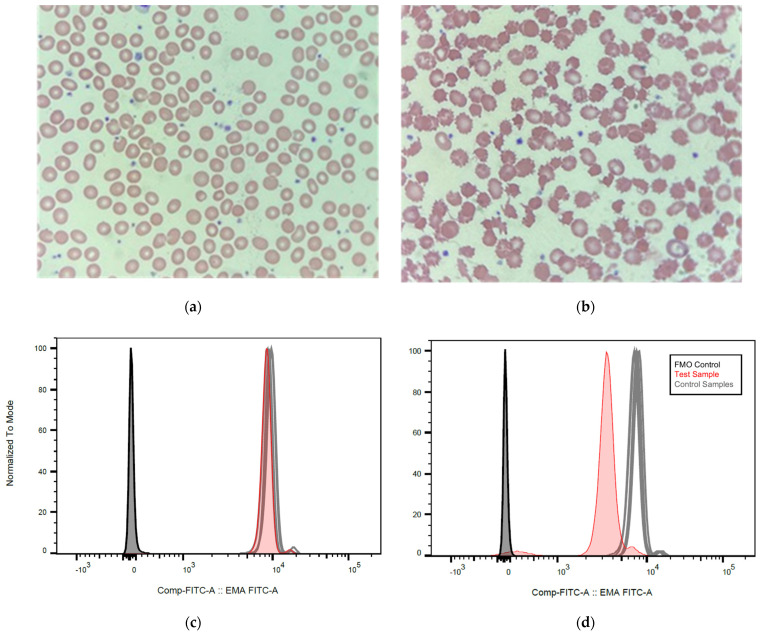

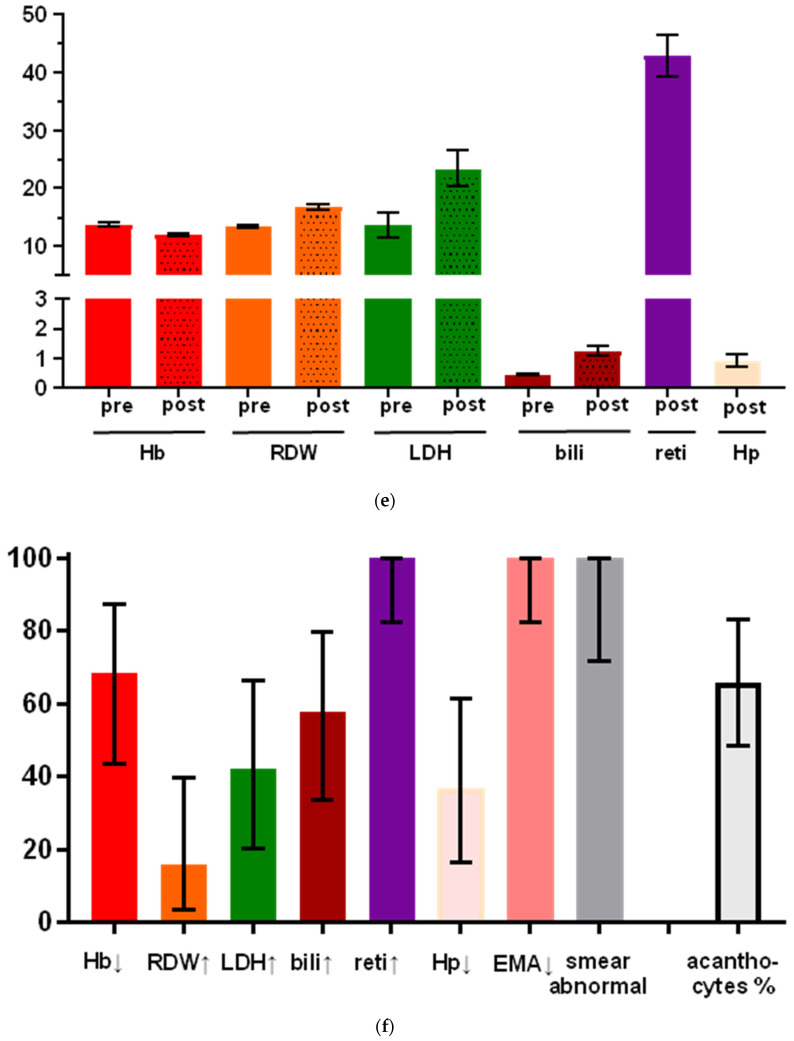
Development of peripheral blood changes after the start of alectinib in ALK+ NSCLC. (**a**) Peripheral blood film of patient 8 (Table 1) at 67× magnification showing normal red cell morphology before alectinib start. (**b**) Peripheral blood film two weeks after alectinib start at 67× magnification showing abnormal red cell morphology with anisocytosis, a predominance of acanthocytes, as well as occasional echinocytes, spherocytes, and rare fragmentocytes (Table 1). (**c**) EMA binding assay of the same patient before therapy with alectinib showing a 10% decrease in EMA fluorescence (reference range <20%). (**d**) In the same patient, six weeks after alectinib start, EMA fluorescence is reduced by 58%. (**e**) Changes in hemoglobin (Hb, in g/dL, *n* = 16), red-cell distribution width (RDW, %, *n* = 17), serum lactate dehydrogenase (LDH, in U/mL/10, *n* = 16), total serum bilirubin (bili, in mg/dL, *n* = 11), blood reticulocytes (reti, %, *n* = 18), as well as levels of serum haptoglobin (Hp, in g/L, *n* = 19) and after start of alectinib in our patients. Statistical comparisons were performed with a paired t-test for parameters with available data before and after alectinib start (Hb, RDW, LDH, bilirubin) or using a t-test comparison vs. the upper or lower limit of the normal range for other parameters (5–15% for reticulocytes, and 0.3–2.0 g/L for haptoglobin). Box plots indicate mean values with their standard errors (from left to right: 13.8 g/dL vs. 12.0 g/dL, 13.4% vs. 16.8%, 136 U/L vs.234 U/L, 0.45 mg/dL vs.1.26 mg/dL, 43% with *p* < 0.0001 compared to the normal range, 0.93 g/L within the normal range of 0.3–2.0 g/L); ns: not statistically significant. (**f**) The percentage of patients with abnormal values for each of the parameters analyzed in this study, according to Table 1. Bars indicate percentages with 95% confidence intervals: 68.4% [43.5–87.4] for reduced serum hemoglobin, 15.8% [3.4–39.6] for increased RDW, 42.1% [20.2–66.5] for increased serum LDH, 57.9% [33.5–79.8] for increased serum bilirubin, 100% [82.4–100] for increased blood reticulocytes, 36.8% [16.3–61.6] for reduced serum haptoglobin, 100% [82.4–100] for reduced EMA binding, and 100% [71.5–100] for the presence of abnormalities in the blood smear, while the mean percentage of acanthocytes in the peripheral blood smear was 65.8% [48.6–83.1].

**Figure 2:**
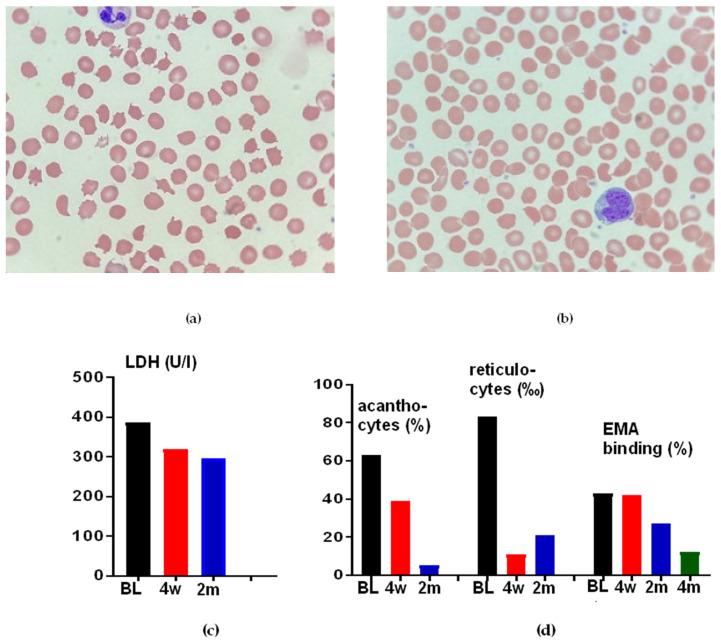

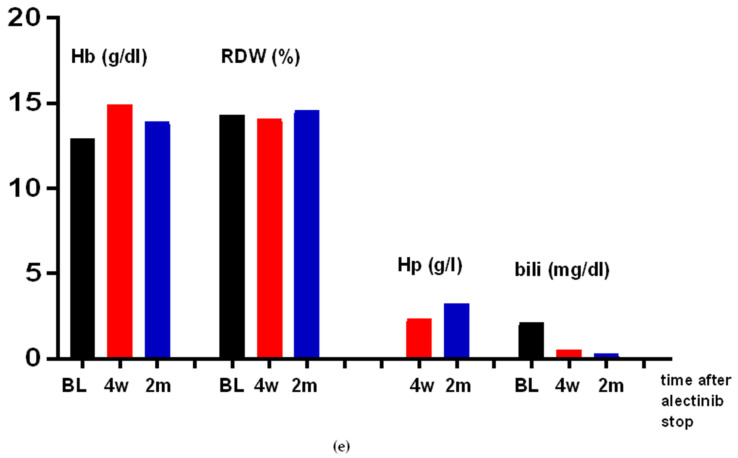
Resolution of erythrocyte changes after alectinib stop in patient 1 (Table 1). (**a**) Peripheral blood film at 100× magnification showing abnormal red cell morphology during therapy with alectinib. (**b**) Blood smear 4 weeks after the switching of therapy to brigatinib at 100× magnification with the return of some normal erythrocytes. (**c**–**e**) Longitudinal resolution or improvement of other changes in the same patient after alectinib stop; BL: baseline; 4 w: 4 weeks after treatment stop; 2 m: 2 months after treatment stop.

The clinical and molecular characteristics of the analyzed patients, as well as the performed hematological tests and their results, are shown in Table 1. A total of nine among the 19 alectinib-treated patients received the drug in the first line, 8/19 in the second line, and 2 (2/19) in the third or subsequent lines. The *ALK* fusion could be typed in 20/24 cases and comprised *EML4-ALK* variant 1 (E13;A20) in 15/20 patients, variant 2 (E20;A20) in 3/20 patients, and variant 3 (E6;A20) in 2/20 patients. Additionally, *TP53* status was available for 22/24 patients, with wild-type results in 18/22 and pathogenic mutations in 4/22 patients (Table 1). Anemia was present in approximately two-thirds (13/19) of analyzed patients and was mostly mild, with the lowest hemoglobin value being 10.2 g/dL in a female patient (Table 1). Routine laboratory tests showed elevated levels of bilirubin in most patients (11/19 or 58%), while LDH was elevated in only 42% (8/19), and haptoglobin was in the lower reference range or not detectable in only approximately one-third of patients (37% or 7/19, Table 1). On the other hand, reticulocytes were increased in all (19/19) studied patients ranging from 19‰ to 83‰ (Table 1). The direct antiglobulin (Coombs) test was negative in 11/11 analyzed patients. Peripheral blood smears showed abnormal morphology of the erythrocytes in all patients (11/11). Most prevalent were acanthocytes (36–100% on average, Figure 1B and Table 1), followed by echinocytes (0–15%), spherocytes (0–10%), dacrocytes (0–5%), and fragmentocytes (0–2.6%). Furthermore, the EMA binding was reduced in all samples (19/19), with negative erythrocytes ranging between 32% and 62% (normal <20%). Of note, the morphologic changes appeared very early after therapy initiation. For example, they were evident in the blood smear of patient #8 after only a few days following the start of alectinib (Figure 1). Changes in the EMA test results followed, and the percentage of negative cells exceeded 20% (the upper-normal limit) 6 weeks after the initiation of alectinib (Figure 2).

The coexistence of acanthocytes and spherocytes together with the reduced EMA binding in most patients was reminiscent of hereditary spherocytosis. This differential diagnosis was particularly relevant for one of our cases (#1 in Table 1), who, aside from hemolysis under alectinib, also showed a paravertebral mass suggestive of extramedullary hematopoiesis in the spine MRI that already existed before the initiation of therapy. However, genetic testing by NGS showed no mutations in *ANK1*, *EPB42*, *SLC4A1*, *SPTA1,* or *SBTB* genes in this and an additional two patients, thus ruling out hereditary spherocytosis in all studied cases (Table 1).

Hematologic changes under alectinib appeared to be specific to this particular drug because they were not observed in patients treated with crizotinib, brigatinib, or lorlatinib (cases 1, 14–18, Table 1). In addition, they appeared to be reversible, as is evident by the prompt resolution in a patient who was switched from alectinib to brigatinib due to tumor progression (case 1, Table 1): the blood smear after 4 weeks showed a significantly decreased number of pathologically altered erythrocytes (10% vs. 40% before the change of therapy, Figure 2). In contrast, the changes in EMA binding assay resolved more slowly, from 43% at the time of the therapy switch to 42% 4 weeks later, 28% 9 weeks after the start of brigatinib (Table 1), and after another 5 weeks, the EMA binding was in the normal range (12%, NB. The patient, meanwhile, received lorlatinib due to a new tumor progression under brigatinib).

Median follow-up from the start of therapy for metastatic ALK+ NSCLC for our alectinib-treated patients (*n* = 19, Table 1) was 57 months (95% confidence interval (CI) 28.1–86.1 months), while their median OS was not reached (3/19 events so far). The median follow-up for PFS under alectinib was 39.5 months (95% CI 29.7–49.3 months), and the median PFS was not reached either (3/19 events). PFS under alectinib showed no significant association with Hb before the start of alectinib (hazard ratio (HR) 1.97, *p* = 0.32), LDH before alectinib start (HR = 0.93, *p* = 0.99), or bilirubin before alectinib start (HR = 0.52, *p* = 0.93). Additionally, PFS under alectinib showed no significant association with Hb under alectinib (HR = 2.71, *p* = 0.066), LDH under alectinib (HR = 1.023, *p* = 0.061), bilirubin under alectinib (HR = 1.15, *p* = 0.85), reticulocytes under alectinib (HR-1.07, *p* = 0.068), or haptoglobin under alectinib start (HR = 1.34, *p* = 0.60). Finally, there were no differences in the aforementioned red blood cell and hemolysis parameters, i.e., Hb before and after alectinib start, LDH before or after alectinib start, bilirubin before and after alectinib start, reticulocytes under alectinib, or haptoglobin under alectinib according to the *EML4-ALK* fusion variant (V3 vs. non-V3) and *TP53* status (wild-type or mutated) of the tumors (all *p*-values >0.10 with unpaired *t*-tests).

## 4. Discussion

Our results show that alectinib induces subclinical hemolysis in practically all patients who receive the drug. Hb decreases are usually mild, resulting in a constellation resembling hereditary spherocytosis based on standard laboratory workup. Genetic testing by NGS can rule out the latter whenever necessary, as exemplified by three patients in our series (Table 1). However, an accurate patient history, along with detailed knowledge of alectinib-induced changes as described here, could resolve the dilemma in many situations.

In order to support differential-diagnostic considerations in these patients, our work provides data on a wide array of parameters and emphasizes the longitudinal course of abnormalities (Table 1). A first important observation is that, although the pattern of abnormalities varies among patients, three changes appear to occur universally under alectinib: abnormal erythrocyte morphology, increased reticulocytes, and decreased EMA binding, each of which was observed in all patients studied (Table 1). Given the high sensitivity of 100%, the combination of these three findings could therefore be used in order to support or exclude the causative role of alectinib in lung cancer patients presenting signs of hemolysis, the differential diagnosis of which can be very broad and includes, for example, warm autoantibodies induced by antibiotics, cytostatics or other anticancer drugs, microangiopathic disease, as well as bone marrow insufficiency [18,19]. Longitudinal analysis of our patients reveals that all elements of the typical diagnostic triad develop with similar kinetics and become readily detectable within the first month (Table 1 and Figure 1). Other aberrations occur less frequently. For example, anemia was present in only 68%, increased bilirubin in 58%, increased LDH in 42%, and decreased haptoglobin in only 37% of patients (Table 1 and Figure 1f). At the mechanistic level, changes of the erythrocyte membrane leading to acanthocytosis under alectinib could presumably be caused by a direct toxic effect of the drug, as bone marrow changes were not observed during the animal testing phase [20], and acanthocytic deformation was detectable in the vast majority of circulating red cells, with an average percentage of 64% among patients in our series (Figure 1f). Furthermore, the infrequent (37% of cases) and only mild (average 0.93 g/mL within the normal range of 0.2–2.0 g/dL) haptoglobin decreases under alectinib suggest that hemolysis occurs predominantly in the extravascular compartment, for example in the spleen, where distorted erythrocytes tend to become trapped and destructed, as has also been described for patients with other forms of acquired severe acanthocytosis, e.g., in the context of liver cirrhosis [21,22]. The presence of extravascular (compared to intravascular) hemolysis also decreases the sensitivity of serum LDH, but not serum bilirubin as a hemolytic marker [23], so the observed pattern of aberrations, with more patients showing increased bilirubin (58%) than increased LDH (42%) and decreased haptoglobin (37%), also strongly suggests extravascular red call destruction in alectinib-treated patients (Table 1). In contrast, the increase in blood reticulocytes depended only on the rate but not the location of red cell loss and was observed in all analyzed patients as proof of ubiquitous hemolysis under treatment with alectinib (Table 1).

All observed erythrocyte changes appear to be alectinib-specific since they were not encountered in patients treated with other ALK inhibitors (including crizotinib, brigatinib, and lorlatinib). Moreover, these abnormalities are reversible since they resolved after alectinib discontinuation (Table 1 and Figure 2). Of note, some alterations, such as reduced EMA binding, may require >1 month to normalize, which is also important to consider during the differential diagnostic workup of lung cancer patients with hemolysis and recent alectinib exposure (Table 1 and Figure 2). It should also be highlighted that erythrocyte indices and hemolysis parameters, such as Hb, LDH, bilirubin, reticulocytes, and haptoglobin, showed no association with the molecular features of the disease, i.e., *EML4-ALK* variant and *TP53* status, or the PFS under alectinib in our patients. Therefore, they lack prognostic value and potential utility as monitoring parameters, in contrast to several genetic markers from blood, for example, ctDNA mutations and copy number alterations, which have recently emerged as very promising disease-surveillance tools [24].

Other authors have recently reported cases of ALK+ NSCLC under alectinib with similar findings [25,26]. The specific strengths of the current study are the relatively large number of patients, including a control group of patients under other ALK TKIs; the wide array of examined hematologic parameters, including longitudinal tracking; the precise quantification of various morphologically abnormal erythrocyte subpopulations; the genetic testing using NGS to rule out spherocytosis; and consideration of patient outcomes as well as the molecular features of the disease. The clinical importance of chronic alectinib-induced hemolysis appears to be limited in the short-term, as the percentage of patients with alectinib-induced grade 3–4 anemia is low, 5% in the ALEX study [5], and no association with PFS or prognostically relevant features, such as the *EML4-ALK* V3 and *TP53* status, was noted in our patients [27,28]. Nonetheless, most ALK+ patients receiving alectinib have a life expectancy exceeding 5 years [9], especially in the absence of high-risk features such as the *EML4-ALK* variant 3 of *TP53* mutations [29,30], so the risk of long-term complications, such as cholelithiasis due to increased serum bilirubin in most cases (58%, Figure 1e), remains unclear at present [31]. Whenever clinically relevant anemia ensues, thorough the knowledge of alectinib-induced hemolytic changes, as presented in this work, becomes crucial to supporting a meticulous differential diagnosis from other mechanisms of anemia in lung cancer [32].

The main limitations of the current study are the relatively small sample size (*n* = 19 for alectinib-treated patients) and the immature survival data with a few events (3/19) so far, which are ultimately due to the rarity and exceptionally good prognosis of the ALK+ disease, as well as due to the prospective nature of this investigation. Nonetheless, the validity of our observations is underscored by the fact that several erythrocyte changes, i.e., increased reticulocytes, decreased EMA binding, and morphologic abnormalities were evident in all (19/19) analyzed patients. Furthermore, the paucity of disease progression or death, despite the relatively long follow-up for PFS (after 39.5 months median follow-up time in only 3/19 events), the lack of any trend for association between PFS and hemolytic parameters, and the putative pathogenesis of hemolysis in all analyzed patients as a direct toxic effect of alectinib, taken together, argue strongly against a potential prognostic or predictive importance for red blood cell aberrations in metastatic ALK+ NSCLC treated with this drug.

## 5. Conclusions

Alectinib induces subclinical hemolysis in practically all treated patients, which is likely caused by acanthocytic changes and the splenic trapping of circulating erythrocytes. This constellation is indistinguishable from hereditary spherocytosis based on standard laboratory tests and resolves after a switch to other ALK TKIs. These changes did not show any association with the efficacy of alectinib or the biology of ALK+ disease in our study but have differential diagnostic implications for any hematologic workup and could potentially carry long-term risks that remain unclear at present.

## Figures and Tables

**Table 1:** Clinical, molecular, and laboratory data. The reference values for each parameter are given in parentheses. Pathologic values are highlighted in bold.

Patient #, Sex	*ALK* Fusion	*TP53* Status	ALK TKI (Line)	Hb (>12–13 g/dL)	LDH (<317 U/L)	Bilirubin (<1 mg/dL)	Haptoglobin (0.3–2.0 g/L)	Reticulocytes (5–15‰)	Blood Smear: A/S/E/D/S/F (%)	RDW (%) (12.9–18.7)	EMA-neg (<20%)	Other Analyses
1, m	V1	wt	Crizotinib Alectinib (2) Brigatinib Lorlatinib Lorlatinib	14.3 **12.6** 14.9 13.9 13.5	270 **386** **319** 296 304	0.2 **2.1** 0.5 0.3 0.4	- **<0.1** 2.35 3.24 3.69	- **83.00** 11.00 **22.00** 14.00	- 63/10/5/5/0.2/0.5 39/10/0/2/0.2/1 5/1/0/0.5/0/0.5	13.2 14.3 14.1 14.6	- **43** **42** **27** 12 *	DAT NGS HbE trait
2, m	n/a	n/a	Pre-alectinib Alectinib (1)	14.6 13.8	189 309	- **1.7**	- **<0.1**	- **40.00**	- 42/7/0/0/0,5/1,5	13.8 15.9	- **43**	DAT NGS
3, m	V1	p.P85 fs *38	Pre-alectinib Alectinib (1)	13.3 **11.8**	227 **357**	0.3 **1.9**	- 0.58	- **57.00**	- 80/1/2/0.1/0/0.8	13.3 16.2	- **46**	DAT, NGS HbE trait
4, f	V1	p.S183 *	Pre-alectinib Alectinib (1)	**11.8** **11.0**	185 239	0.3 0.8	0.58	**37.00**	- 91/1/5/1/1/0.5	13.6 16.5	**57**	DAT
5, m	n/a	wt	Pre-alectinib Alectinib (2)	13.6 **12.9**	290 243	0.5 **1.1**	1.74	**36.00**	- 46/7/0/0.1/0/1.5	13.3 16.7	**42**	
6, m	V1	wt	Pre-alectinib Alectinib (1)	15.4 **11.0**	295 **431**	0.8	**0.27**	**42.00**		13.4 **19.6**	**50**	
7, f	V1	wt	Pre-alectinib Alectinib (2)	13.7 12.4	201 **354**	0.3 **1.1**	3.0	**40.00**	- 70/0/15/0.2/0/1	12.7 19.2	**48**	DAT
8, f	V1	wt	Pre-alectinib Alectinib (1)	12.5 **10.9**	190 **398**	0.5 0.9	2.86	**48.00**	- 91/0/5/0.5/0.1/2	14.0 **20.2**	10 58 ***	DAT
9, f	V1	wt	Pre-alectinib Alectinib (2)	12.5 **10.6**	173 **358**	**1.0**	**<0.1**	**48.00**	- 36/2/0/0/0/2.6	13.6 16.4	**39**	
10, m	V1	wt	Pre-alectinib Alectinib (1)	15.2 **12.4**	170 263	0.6 **1.4**	0.4	**34.00**		12.9 18.5	**32**	DAT
11, f	n/a	n/a	Pre-alectinib Alectinib (1)	**11.1** **10.2**	202 302	0.4 0.6	2.21 0.98	**57.00**		14.6 16.8	0 62 ****	
12, f	V1	wt	Pre-alectinib Alectinib (5)	14.2 12.5	245 **332**	0.5 0.6	0.89	**28.00**	- **39**/0/**9**/**4**/0/0	13.2 14.9	**47**	DAT
13, m	V1	wt	Pre-alectinib Alectinib (1)	15.2 **12.5**	244 278	0.8 **2.9**	1.09	**51.00**	- **100**/0/0/0/0/0	13.8 17.4	48	DAT
14, m	n/a	wt	Brigatinib	15.3	266	0.5	0.78	12.00		13.6	8	
15, m	V2	p.H193D	Brigatinib	**12.9**	197	0.2	1.77	15.00		13.5	0	
16, m	V3	wt	Lorlatinib	14.9	200	0.3	2.0	**20.00**		14.6	5	
17, f	V1	wt	Crizotinib	**10.2**	260	0.4				14.4	0	
18, m	V3	p.R213Q	Brigatinib Alectinib (2)	15.0 15.4	308 **444**	0.2 0.3	3.19 2.77	15.00 **37.00**		15.0 15.7	17 50 **	DAT
19, m	V1	p.spl?	Pre-alectinib Alectinib (2)	14.1 13.9	263 232	0.5 0.9	0.88 **0.14**	**22.00** **25.00**		14.1 15.0	7 39 ** 54 **	
20, m	V1	wt	Pre-alectinib Alectinib (0)	14.4 14.1	191 225	0.5 0.6	1.02	**19.00**		13.6 13.9	1 40 **	
21, w	V1	wt	Crizotinib Alectinib (2)	12.0 **10.7**	248 145	0.3 **1.2**	**0.1**	**44.00**		13.3 14.9	60 **	
22, m	V2		Lorlatinib	**12.9**	209	0.3	3.08	**17.00**		15.3	0	
23, f	V2	wt	Alectinib (3)	**11.0**	264	**1.8**	**0.26**	**63.00**	-	17.3	**52**	
24, f	V1	wt	Alectinib (2)	**9.1**	197	**3.4**	0.8	**34.00**	88/6/5/0/0/1	**21.0**	**57**	DAT
	% (*n*) of alectinib-treated pts. with abnormalities			**68.4%** **(13/19)**	**42.1%** **(8/19)**	**57.9%** **(11/19)**	**36.8%** **(7/19)**	**100%** **(19/19)**	**100%** **(11/11)**	**15.8%** **(3/19)**	**100%** **(19/19)**	

m, male; f, female; n/a: not available; ALK, anaplastic lymphoma kinase; TKI: inhibitor; wt: wild-type; mut: mutated; Hb: hemoglobin (reference f: 12–15, m: 13–17); LDH, lactate dehydrogenase; EMA, eosin-5-maleimide; pts: patients; DAT: direct antiglobulin test (direct Coombs test); NGS: next-generation sequencing for hereditary spherocytosis (s. Methods); A/S/E/D/S/F: acanthocytes/spherocytes/echinocytes/dacrocytes/stomatocytes/fragmentocytes. * EMA binding returned to normal 16 weeks after the end of therapy with alectinib; decreased EMA binding was found after 2 weeks (**) or 6 weeks (***), or two months (****) of therapy with alectinib.

## Data Availability

Data analyzed in this study are available upon reasonable request.

## References

[1] Wang M., Herbst R.S., Boshoff C. (2021). Toward Personalized Treatment Approaches for Non-small-Cell Lung Cancer. Nat. Med..

[2] Hanna N.H., Robinson A.G., Temin S., Baker S., Brahmer J.R., Ellis P.M., Gaspar L.E., Haddad R.Y., Hesketh P.J., Jain D. (2021). Therapy for Stage IV Non-Small-Cell Lung Cancer With Driver Alterations: ASCO and OH (CCO) Joint Guideline Update. J. Clin. Oncol..

[3] Planchard D., Popat S., Kerr K., Novello S., Smit E.F., Faivre-Finn C., Mok T.S., Reck M., van Schil P.E., Hellmann M.D. (2018). Metastatic Non-small Cell Lung Cancer: Esmo Clinical Practice Guidelines for Diagnosis, Treatment and Follow-up. Ann. Oncol..

[4] Planchard D., Popat S., Kerr K., Novello S., Smit E.F., Faivre-Finn C., Mok T.S., Reck M., van Schil P.E., Hellmann M.D. ESMO Clinical Practice Living Guidelines—Metastatic Non-Small-Cell Lung Cancer. https://www.esmo.org/guidelines/lung-and-chest-tumours/clinical-practice-living-guidelines-metastatic-non-small-cell-lung-cancer.

[5] Peters S., Camidge D.R., Shaw A.T., Gadgeel S., Ahn J.S., Kim D.-W., Ou S.-H.I., Pérol M., Dziadziuszko R., Rosell R. (2017). Alectinib versus Crizotinib in Untreated ALK-Positive Non-Small-Cell Lung Cancer. N. Engl. J. Med..

[6] Camidge D.R., Kim H.R., Ahn M.-J., Yang J.C.-H., Han J.-Y., Lee J.-S., Hochmair M.J., Li J.Y.-C., Chang G.-C., Lee K.H. (2018). Brigatinib versus Crizotinib in ALK-Positive Non-Small-Cell Lung Cancer. N. Engl. J. Med..

[7] Shaw A.T., Bauer T.M., de Marinis F., Felip E., Goto Y., Liu G., Mazieres J., Kim D.-W., Mok T., Polli A. (2020). First-Line Lorlatinib or Crizotinib in Advanced ALK-Positive Lung Cancer. N. Engl. J. Med..

[8] Gristina V., La Mantia M., Iacono F., Galvano A., Russo A., Bazan V. (2020). The Emerging Therapeutic Landscape of ALK Inhibitors in Non-Small Cell Lung Cancer. Pharmaceuticals.

[9] Mok T., Camidge D.R., Gadgeel S.M., Rosell R., Dziadziuszko R., Kim D.-W., Pérol M., Ou S.-H.I., Ahn J.S., Shaw A.T. (2020). Updated Overall Survival and Final Progression-Free Survival Data for Patients with Treatment-Naive Advanced ALK-Positive Non-Small-Cell Lung Cancer in the ALEX Study. Ann. Oncol..

[10] Ly A.C., Olin J.L., Smith M.B. (2018). Alectinib for advanced ALK-positive non-small-cell lung cancer. Am. J. Health. Syst. Pharm..

[11] Travis W.D., Brambilla E., Nicholson A.G., Yatabe Y., Austin J.H.M., Beasley M.B., Chirieac L.R., Dacic S., Duhig E., Flieder D.B. (2015). The 2015 World Health Organization Classification of Lung Tumors: Impact of Genetic, Clinical and Radiologic Advances Since the 2004 Classification. J. Thor. Oncol..

[12] Volckmar A.-L., Leichsenring J., Kirchner M., Christopoulos P., Neumann O., Budczies J., Morais de Oliveira C.M., Rempel E., Buchhalter I., Brandt R. (2019). Combined Targeted DNA and RNA Sequencing of Advanced NSCLC in Routine Molecular Diagnostics: Analysis of the First 3000 Heidelberg Cases. Int. J. Cancer.

[13] Tate J.G., Bamford S., Jubb H.C., Sondka Z., Beare D.M., Bindal N., Boutselakis H., Cole C.G., Creatore C., Dawson E. (2019). COSMIC: The Catalogue Of Somatic Mutations In Cancer. Nucleic Acids Res..

[14] Arora R.D., Dass J., Maydeo S., Arya V., Radhakrishnan N., Sachdeva A., Kotwal J., Bhargava M. (2018). Flow Cytometric Osmotic Fragility Test and Eosin-5’-Maleimide Dye-Binding Tests Are Better than Conventional Osmotic Fragility Tests for the Diagnosis of Hereditary Spherocytosis. Int. J. Lab. Hematol..

[15] Russo R., Marra R., Rosato B.E., Iolascon A., Andolfo I. (2020). Genetics and Genomics Approaches for Diagnosis and Research into Hereditary Anemias. Front. Physiol..

[16] Ma X., Nussbaum N.C., Magee K., Bourla A.B., Tucker M., Bellomo L., Bennette C. (2019). Comparison of Real-World Response Rate (rwRR) to RECIST-Based Response Rate in Patients with Advanced Non-small Cell Lung Cancer (aNSCLC). Ann. Oncol..

[17] Bartlett C.H., Mardekian J., Cotter M., Huang X., Zhang Z., Parrinello C.M., Abernethy A.P., Koehler M. (2018). Concordance of Real World Progression Free Survival (PFS) on Endocrine Therapy as First Line Treatment for Metastatic Breast Cancer Using Electronic Health Record with Proper Quality Control versus Conventional PFS from a Phase 3 Trial. Cancer Res..

[18] Spivak J.L. (1994). Cancer-Related Anemia: Its Causes and Characteristics. Semin. Oncol..

[19] Leaf R.K., Ferreri C., Rangachari D., Mier J., Witteles W., Ansstas G., Anagnostou T., Zubiri L., Piotrowska Z., Oo T.H. (2019). Clinical and Laboratory Features of Autoimmune Hemolytic Anemia Associated with Immune Checkpoint Inhibitors. Am. J. Hematol..

[20] Therapeutic Goods Administration, Australian Department of Health AusPAR Alecensa Alectinic hydrochloride Roche Products Pty Limited PM-2015-04677-1-4. https://www.tga.gov.au/sites/default/files/auspar-alectinib-hydrochloride-171127.pdf.

[21] Privitera G., Meli G. (2016). An Unusual Cause of Anemia in Cirrhosis: Spur Cell Anemia, a Case Report with Review of Literature. Gastroenterol. Hepatol. Bed Bench.

[22] Marks E.I., Ollila T.A. (2019). Acanthocytosis Causing Chronic Hemolysis in a Patient with Advanced Cirrhosis. Blood.

[23] Barcellini W., Fattizzo B. (2015). Clinical Applications of Hemolytic Markers in the Differential Diagnosis and Management of Hemolytic Anemia. Dis. Markers.

[24] Dietz S., Christopoulos P., Yuan Z., Angeles A.K., Gu L., Volckmar A.-L., Ogrodnik S.J., Janke F., Fratte C.D., Zemojtel T. (2020). Longitudinal Therapy Monitoring of ALK-Positive Lung Cancer by Combined Copy Number and Targeted Mutation Profiling of Cell-Free DNA. EBioMedicine.

[25] Kuzich J.A., Heynemann S., Geoghegan N., Evelyn C., O’Mahoney S., Wilson S., Campbell J., Rogers K., Solomon B., Westerman D. (2021). Alectinib Induces Marked Red Cell Spheroacanthocytosis in a Near-Ubiquitous Fashion and Is Associated with Reduced Eosin-5-Maleimide Binding. Pathology.

[26] Gullapalli V., Xu W., Lewis C.R., Anazodo A., Gerber G.K. (2021). A Multi-Centre Case Series of Alectinib-Related Erythrocyte Membrane Changes and Associated Haemolysis. J. Hematopathol..

[27] Christopoulos P., Kirchner M., Bozorgmehr F., Endris V., Elsayed M., Budczies J., Ristau J., Penzel R., Herth F.J., Heussel C.P. (2019). Identification of a Highly Lethal V3+ TP53+ Subset in ALK+ Lung Adenocarcinoma. Int. J. Cancer.

[28] Christopoulos P., Kirchner M., Endris V., Stenzinger A., Thomas M. (2018). EML4-ALK V3, Treatment Resistance, and Survival: Refining the Diagnosis of ALK+ NSCLC. J. Thor. Dis..

[29] Elsayed M., Christopoulos P. (2021). Therapeutic Sequencing in ALK+ NSCLC. Pharmaceuticals.

[30] Christopoulos P., Budczies J., Kirchner M., Dietz S., Sültmann H., Thomas M., Stenzinger A. (2019). Defining Molecular Risk in ALK+ NSCLC. Oncotarget.

[31] Bates G.C., Brown C.H. (1952). Incidence of Gallbladder Disease in Chronic Hemolytic Anemia (Spherocytosis). Gastroenterology.

[32] Pirker R., Wiesenberger K., Pohl G., Minar W. (2003). Anemia in Lung Cancer: Clinical Impact and Management. Clin. Lung Cancer.

